# AA-RGTCN: reciprocal global temporal convolution network with adaptive alignment for video-based person re-identification

**DOI:** 10.3389/fnins.2024.1329884

**Published:** 2024-03-25

**Authors:** Yanjun Zhang, Yanru Lin, Xu Yang

**Affiliations:** ^1^School of Cyberspace Science and Technology, Beijing Institute of Technology, Beijing, China; ^2^School of Integrated Circuits and Electronics, Beijing Institute of Technology, Beijing, China; ^3^School of Computer Science and Technology, Beijing Institute of Technology, Beijing, China

**Keywords:** convolutional neural network, video person re-identification, image recognition, temporal modeling, frame alignment

## Abstract

Person re-identification(Re-ID) aims to retrieve pedestrians under different cameras. Compared with image-based Re-ID, video-based Re-ID extracts features from video sequences that contain both spatial features and temporal features. Existing methods usually focus on the most attractive image parts, and this will lead to redundant spatial description and insufficient temporal description. Other methods that take temporal clues into consideration usually ignore misalignment between frames and only focus on a fixed length of one given sequence. In this study, we proposed a Reciprocal Global Temporal Convolution Network with Adaptive Alignment(AA-RGTCN). The structure could address the drawback of misalignment between frames and model discriminative temporal representation. Specifically, the Adaptive Alignment block is designed to shift each frame adaptively to its best position for temporal modeling. Then, we proposed the Reciprocal Global Temporal Convolution Network to model robust temporal features across different time intervals along both normal and inverted time order. The experimental results show that our AA-RGTCN can achieve 85.9% mAP and 91.0% Rank-1 on MARS, 90.6% Rank-1 on iLIDS-VID, and 96.6% Rank-1 on PRID-2011, indicating we could gain better performance than other state-of-the-art approaches.

## 1 Introduction

Person re-identification (Leng et al., 2020) aims at retrieving particular pedestrians across several non-overlapped cameras. It becomes a hot research topic due to its special role in some advanced applications. Compared with a single image that only has spatial features, the video contains both spatial and temporal features, which could help us to extract more robust features. Aiming to make up for the visual limitations of fixed cameras and can be combined with pedestrian detection / pedestrian tracking technology, Re-ID technologies can be widely used in intelligent video surveillance, intelligent security, and other fields.

For example, in the field of intelligent security, Re-ID technologies can be used to assist the search for traces of suspects from surveillance and can also be used to build an intelligent search system to help find lost children or the elderly. Re-ID technologies can also be applied to smart business, for example, by analyzing customer behavior trajectory in large shopping malls, to more clearly build the profile of users, or support the construction of unmanned supermarkets. The development of personal intelligence, such as home robotics technology, also needs the support of Re-ID technologies.

Most relative works (Liu et al., 2015; Xu et al., 2017; Breckon and Alsehaim, 2021) can be summarized as two main steps: spatial feature extraction and temporal feature aggregation. CNN will be used as a spatial feature extractor for each frame, and the generated frame-level features will be sent to the aggregation part to produce a video-level feature. For the aggregation part, global average pooling (Gao and Nevatia, 2018) can achieve a good result, so some works focus on spatial feature extraction and achieve great results, but some original datasets have obvious deficiencies such as misalignment among different frames and occlusion. Using a simple global average pooling will let the network treat bad frames as important as good frames and fuse unexpected parts into the final description due to the misalignment. To address these drawbacks, many methods have been proposed to replace the global average pooling with attention-based modules (Liu et al., 2017) or partition-based modules (Chen and Yang, 2021; Wei et al., 2022), and they really improve final results.

**Figure 1:**
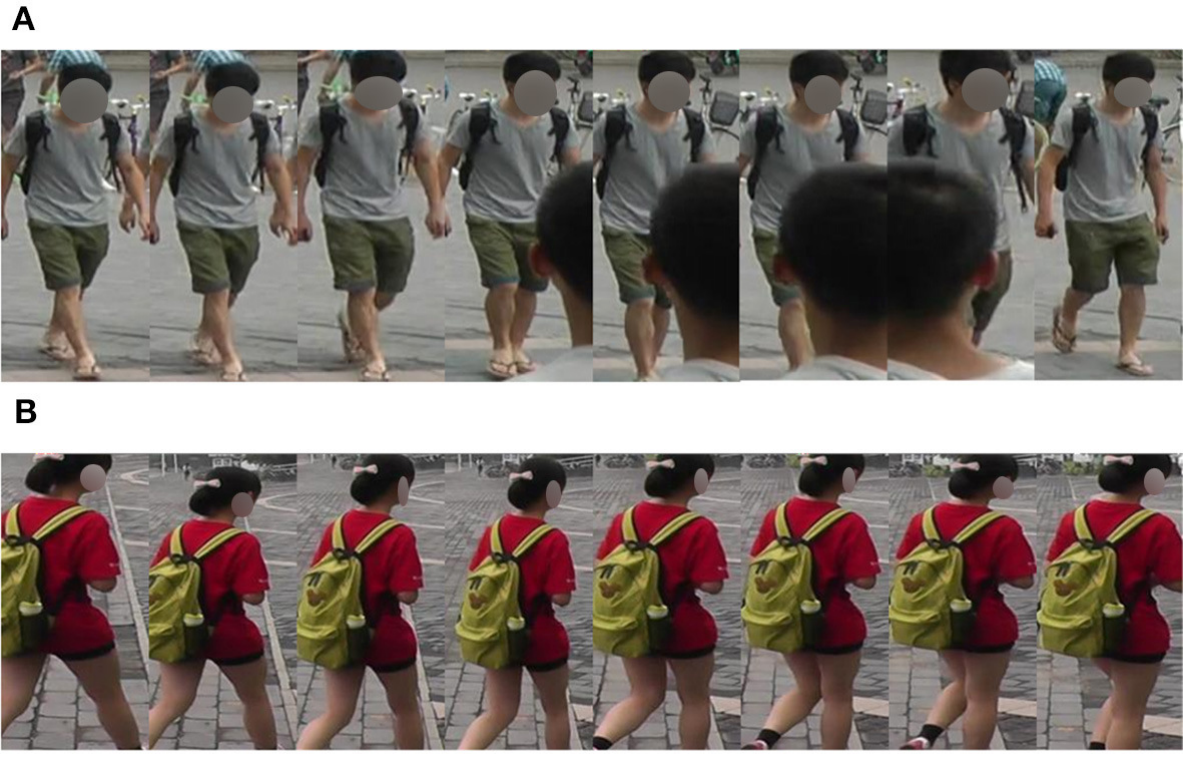
Examples to show the necessity of proper periods of time for different sequences. **(A)** A shorter period is preferred. **(B)** A longer period is preferred.

However, the aggregation methods mentioned above are closer to the way of finding missing or distorted parts in other frames and repairing them, which means temporal clues are not fully used. Therefore, many methods focus on digging the useful temporal clues along the timing order, but most of them only focus on a fixed period of time like Hou et al. (2021b) which focuses on relatively long-term clues and Chen (2020) focuses on some short-term clues. There are also some methods that try to focus on both long and short term (Li et al., 2019a), but they cannot give proper concern for different periods of time. This will lead to a lower performance since we need to focus on different periods of time for different sequences. For instance, given two sequences as shown in Figure 1, it is clear that we want to use a shorter period for sequence (a) and a longer period for sequence (b) to generate robust features.

Previous methods mentioned above focusing on various temporal aggregation approaches achieve great results, but the aggregation module is at the end of the spatial feature extraction module, meaning many low-level temporal features at the beginning part of CNN cannot be directly fused into the final representation. For the same reason, the features that the temporal aggregation module used focus on discriminative local features and ignore global features which also have abundant useful information since CNN will focus on the same position for similar inputs.

Based on the above discussion, we proposed the Reciprocal Global Temporal Convolution Network with Adaptive Alignment (AA-RGTCN) for video-based person re-identification. The network contains two main modules. One is Adaptive Alignment(AA) module, and another is the Reciprocal Global Temporal Convolution Network (RGTCN) part. AA module is designed to align each frame along the time direction, so the following network could start with a better temporal description. It will look through the horizontal and vertical directions and generate offsets for each frame in both two directions and use bilinear polarization to shift them. The proposed AA module will be added before any temporal modeling parts. Then, the RGTCN module will extract temporal features along both forward and inverted time order with different time intervals. This module is inserted into ResNet-50 Bottleneck modules and will be added into different stages to generate both high-level and low-level temporal features. In this way, our proposed network could be able to obtain discriminative features and conquer the effect of misalignment.

The main contributions of our study are listed as follows:

- We propose a novel Reciprocal Global Temporal Convolution Network (RGTCN), which could exploit temporal clues with different intervals and directions efficiently.
- Furthermore, we introduce the Adaptive Alignment (AA-RGTCN) to align each frame to generate more robust temporal features for the RGTCN.
- We compared our approach with other state-of-the-art methods on three popular datasets: MARS, iLIDS-VID, and PRID-2011. The results show that our approach could achieve remarkable performance.

In the following, we first discuss related work in Section 2; then, we will describe the proposed method in Section 3, including two subsections that introduce the AA module and RGTCN module, respectively. In Section 4, we show the experiments discuss the result, and conduct the ablation study. Finally, we conclude the article and discuss the limitations and future directions in the Conclusion.

## 2 Related works

### 2.1 Video-based person re-identification

With the development of deep learning, many areas have gained great success including person re-identification. At first, most works extract features for each frame by CNN backbones such as ResNet-50 and use global average pooling or some simple weighted method to fuse them like Zhao et al. (2019); Breckon and Alsehaim (2021) and Zheng et al. (2016). It helps them to achieve significant results, but they merely use temporal clues to build up a discriminative representation. For temporal clues, many works try to import existing methods, such as adding optical flow to achieve an auxiliary recognition (Simonyan and Zisserman, 2014; McLaughlin et al., 2016), using 3D convolution to model temporal features (Li et al., 2019a; Wu et al., 2019; Aich et al., 2021), introducing RNN to find temporal clues between frame-level representations (Xu et al., 2017; Eom et al., 2021) or modifying non-local block into their proposed framework (Hou et al., 2021b). They really achieve better results compared with methods that only use spatial clues, but some methods such as RNN and 3D convolutions without optimization may not show their strength in such kinds of tasks (Gao and Nevatia, 2018). The rest of them do not fully consider the influence of the variable characteristic of temporal clues.

### 2.2 Temporal feature modeling

Thus, many recent works focus on suitable temporal modeling methods in the video-based person re-identification domain. Li et al. (2019b) use dilation convolution with different strides to extract temporal features via different time intervals and then use a self-attention module to model discriminative features. Yiheng et al. (2019) proposed the Refining Recurrent Unit to fix occlusion and blurred area for a single frame. To better model temporal features, it proposed an integration module to take fused spatial-temporal clues and motion context into consideration. Liu et al. (2021) proposed two modules to gather both high correlation and low correlation features along forward and backward time directions. Fu et al. (2019) split each frame-level feature horizontally and weigh them by a joint spatial-temporal attention module to generate a more robust video-level feature. All the methods discussed above use suitable temporal modeling methods and achieve great success, but they only utilize the high-level spatial features which are the output of the end of a CNN to generate relative temporal clues, ignoring that many low-level spatial features could also contain useful information.

To fully utilize temporal features, many methods use multi-branch networks like Li et al. (2019a); Chen et al. (2017) and Feichtenhofer et al. (2019) have been proposed. This kind of method has more than one CNN branch to gather different types of features. Li et al. (2019a) add one 3D convolution branch to extract temporal features and fuse with the original spatial features. Chen et al. (2017) add branches with different scales to extract features under different resolutions and fuse them. Feichtenhofer et al. (2019) focusing on the difference of motion speed proposed a two-branch network. The slow branch is designed to find spatial clues, and the fast one is for temporal clues. However, this kind of network usually has a huge number of parameters and operations compared with the single-branch network which has similar structures since each branch in multi-branch networks is almost a full CNN.

According to the above discussion, we proposed an architecture to conquer these deficiencies. Specifically, we use one branch structure and could fuse spatial and temporal features in different feature levels along both forward and inverted time orders across different time intervals. To gather a better representation, we also align each frame before we start to extract any temporal clues.

## 3 Proposed method

In this section, we will introduce the proposed Reciprocal Global Temporal Convolution Network with Adaptive Alignment(AA-RGTCN). First, we give an overview of the proposed network. The two components we designed will be discussed in the following subsections.

### 3.1 Overview

**Figure 2:**
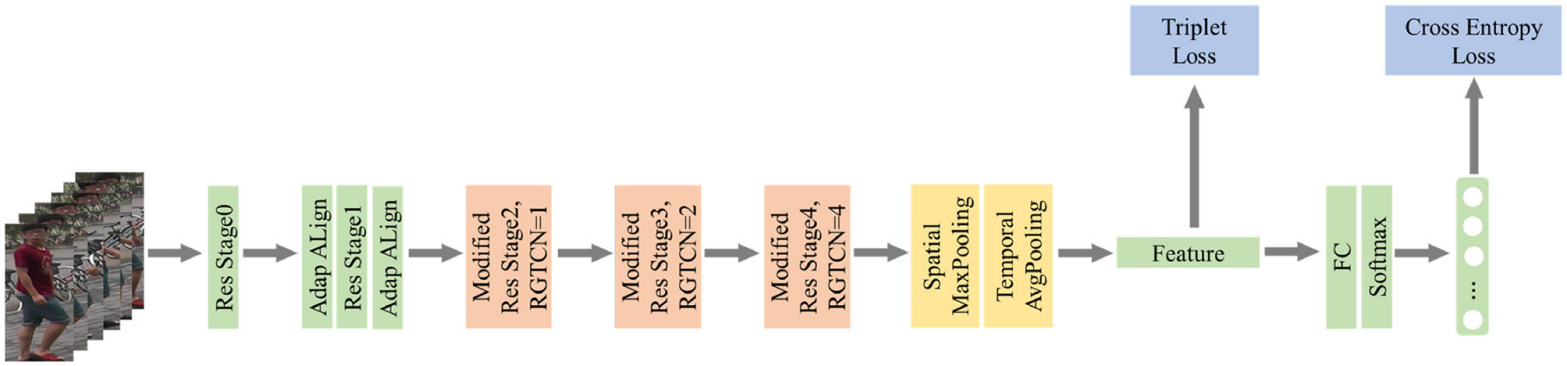
Overview of the total structure.

**Figure 3:**
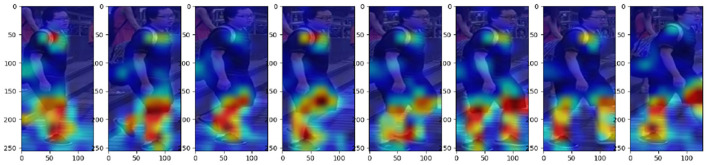
Class activation heatmap of the result.

The overall architecture of our network is shown in Figure 2. We use ResNet-50 pre-trained on ImageNet as our backbone and add two modules called Adaptive Alignment and Reciprocal Global Temporal Convolution Network to help extract more robust temporal features. Specifically, we add two AA modules before and after the Resnet Stage1 to shift each frame to reduce the deficiency of position changes of a certain person's center of gravity. Then, we add RGTCN blocks into all the following stages with different dilation strides to obtain different time interval features along two directions: forward and backward. After the ResNet-50, we use Maxpooling on spatial dimension and Avgpooling on temporal dimension to generate a final video-level representation. During training, an additional FC layer will be added to generate the crossentropy loss. This layer will be removed during inference, and the feature obtained by pooling will be used to calculate the distance. The visualizing heatmaps of class activation of our approach are shown in Figure 3. This heatmap shows our method successfully focuses on the discriminative temporal features such as the legs' moving pace and style of a pedestrian as well as the spatial clues such as the backpack.

### 3.2 Adaptive alignment module

**Figure 4:**
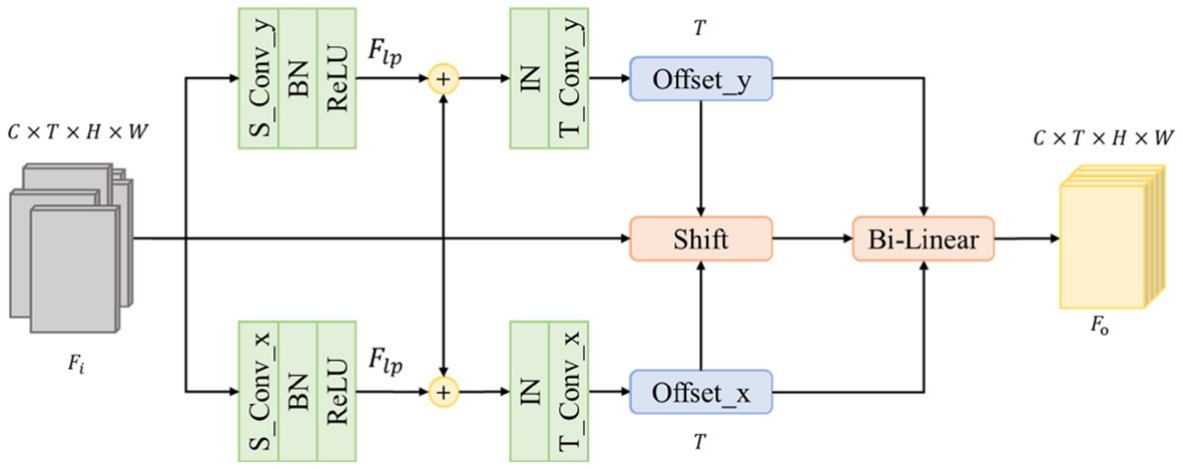
Structure of Adaptive Alignment module.

Most existing methods use datasets to generate representation directly. But the dataset is not perfect, misalignment and mistakes during cropping exist widely in many datasets. This will cause the vibration of the person's center of gravity and further cause the vibration to become one part of the representation, leading to an unreliable result. To address this problem, we propose the Adaptive Alignment Module(AA) to shift each frame and make a more distinguishable feature to the following part. Figure 4 shows the structure of the proposed module.

Given a sequence with T frames, we note it as $F={\left\{F^{n}\right\}}_{t=1}^{T}$ and each frame as $F_{i}\in {\mathbb{R}}^{C*H*W}$ where C, H, and W represent the number of channels, height, and weight, respectively. We split the horizontal and vertical directions into two branches to avoid influence from another direction. In this way, we could obtain the shifting offset of each direction. To obtain more accuracy offsets, we add an additional spatial convolution layer to abstract large-scale position shifting information *F*_*lp*_ such as the shifting of a backpack since the original *F*_*i*_ only contains small-scale position shifting information such as the shifting of the stripe on the backpack. The kernel size of the additional spatial convolution layer is 7 in the required direction and 1 in other directions. Then, the original *F*_*i*_ will be added to *F*_*lp*_ to generate *F*_*p*_ which contains both small-scale and large-scale position information. Notes the spatial convolution as $C_{t}$, the $F_{p}\in {\mathbb{R}}^{C*H*W}$ is as follows Equation 1:


(1) $$\begin{array}{l} F_{p}=F_{i}+C_{t}\left(F_{i}\right) \end{array}$$

Then, we use an Instance Norm to normalize *F*_*p*_ for each frame instead of the whole batch. It helps the following layers find more accurate moving offsets individually. The final offsets are generated by a temporal convolution layer that observes the same position of all frames. Each frame will have its own offsets along two directions, so the final output channel number is determined by *T*.

After getting offsets, we shift each frame to its desired position and further use a bilinear interpolation module to generate the final result which has the same shape of *F*_*i*_.

### 3.3 Reciprocal global temporal convolution network

For person re-identification, classic temporal modeling methods such as RNN may not work well. 3D CNN is an alternative choice since it fits the human's intuition of video perception, but 3D CNN has too many parameters and operations compared with a 2D CNN that has the same structure, and like the method using RNN, 3D convolution cannot achieve a great result either (Gao and Nevatia, 2018). Luckily, some modified 2D CNN structures could also be used to generate temporal features, such as TCN (Bai et al., 2018). The main idea of this kind of CNNs is to use dilation convolution along the time direction. Considering images have abundant spatial and meaningful information along both forward and inverted order compared with a 1-D temporal sequence, we build up the Reciprocal Global Temporal Convolution Network(RGTCN) module to extract these features.

**Figure 5:**
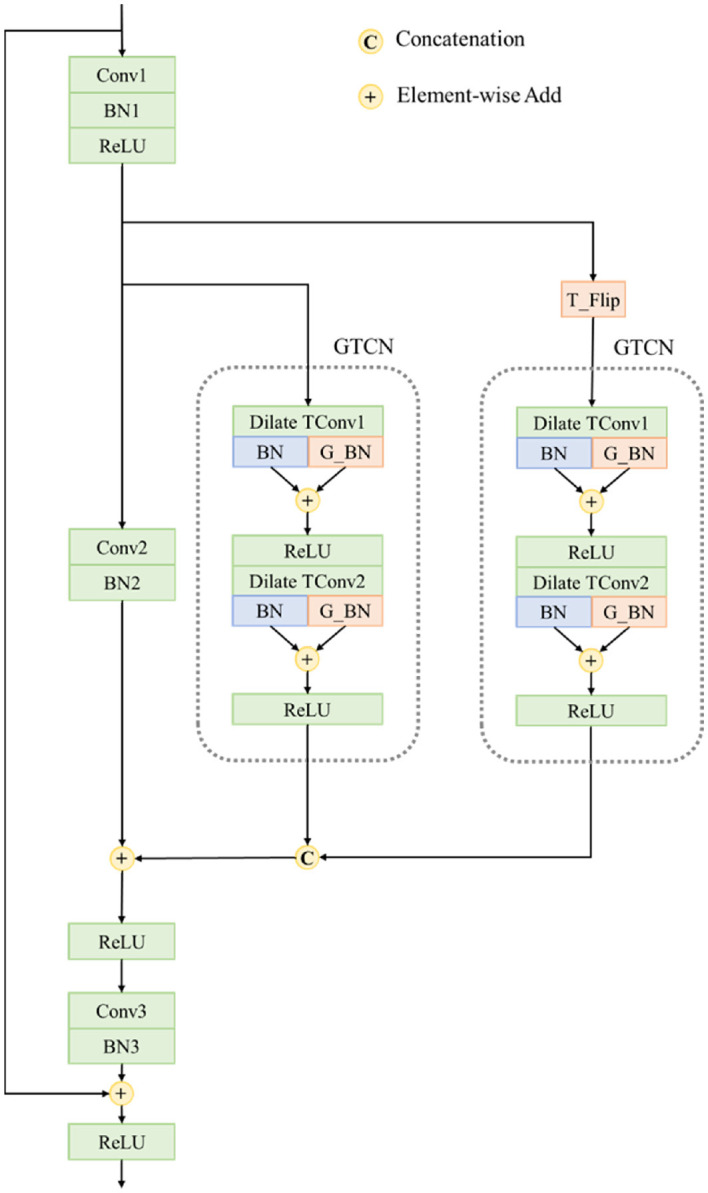
Structure of the Reciprocal Global Temporal Convolution Network. The detail structure of GTCN module is also shown.

First, we design a basic block for temporal modeling, which is surrounded by gray dotted lines shown in Figure 5 and we note this block as Global Temporal Convolution Network(GTCN) block because it can obtain temporal features across different time lengths with both local and global information. It is composed of two dilate temporal convolutions: the normal Batch Normalization layer and the global Batch Normalization layer. Specifically, the two dilate temporal convolutions Dilate TConv1 and Dilate TConv2 have the same kernel size which is 2*1*1 along time, height, and width, respectively, and the stride of dilation is different according to the current Stage in ResNet-50. After Dilate TConv2, the number of output channels will be compressed to half of the number of input channels. The feature obtained by each Dilate TConv will be sent to a normal Batch Norm layer and a global Batch Norm layer at the same time to generate a local description and a global description. These two descriptions will be added to generate a final feature. The global Batch Norm will perform a global average pooling and a normal Batch Norm to generate the final global description. Assuming the feature obtained by Dilate TConv1 is $F_{m}\in {\mathbb{R}}^{C*T*H*W}$, the global average pooling will generate a feature $F_{g}\in {\mathbb{R}}^{C*T*1*1}$ Equation 2:


(2) $$\begin{array}{l} F_{g}=\frac{\overset{H,W}{\underset{h,w=0}{\sum }}F_{m}}{H*W} \end{array}$$

We note the normal Batch Norm as B and the Batch Norm inside the global BN as $B_{g}$, ReLU as R, so the final result *F*_*o*_ is as follows Equation 3:


(3) $$\begin{array}{l} F_{o}=R\left(B\left(F_{m}\right)+B_{g}\left(F_{g}\right)\right) \end{array}$$

The GTCN block is inserted into the ResNet-50 Bottleneck modules as shown in Figure 5, where the T_Flip will invert the original time order. In each Bottleneck module, two GTCNs will be added to simultaneously extract both normal and inverted time order temporal features. Obtained features will be concatenated along the channel and added with spatial features. In this way, we can generate a fused feature containing various spatial-temporal clues. The modified Bottleneck module is noted as the RGTCN module since it further contains global temporal features along two timing orders.

In different Stages of ResNet-50, the stride of dilation will be different to model temporal features from short term to long term. In Stage2, the origin Bottleneck blocks will be replaced by RGTCN Bottleneck with 1 dilation stride. Similarly, in Stage3, the stride is 2, and in Stage4, the stride is 4. Since the total number of frames is 8, after Stage4, we could generate a temporal description containing the information of the whole sequence as well as any sub-sequence which has two and four frames.

## 4 Experiment

### 4.1 Datasets and evaluation protocols

We use three widely used datasets to evaluate our model, which are MARS (Zheng et al., 2016), iLIDS-VID (Wang et al., 2014), and PRID-2011 (Hirzer et al., 2011). MARS is a large-scale dataset that contains 1,261 identities and 20,715 video sequences. All video sequences are captured under six different cameras. PRID-2011 dataset contains 200 identities and 400 video sequences captured by 2 different cameras, and the length of video sequences is from 5 to 675 frames. Following previous work (Wang et al., 2014), we only use video sequences which is longer than 21 frames. iLIDS-VID dataset contains 300 identities and 600 video sequences, and 2 non-overlapped cameras are used to capture all the sequences.

For evaluation, we follow previous work and use Cumulative Matching Characteristic (CMC) and mean Average Precision (mAP) as our evaluation metrics. We only report Rank-1 while evaluating on iLIDS-VID and PRID-2011 datasets because only one correct match is in the gallery.

### 4.2 Implementation details

We use Pytorch as our implementation framework. We randomly sample eight frames with a stride of four to generate training sequences. The training batch size is 64 and contains 16 identities, each identity with 4 video sequences. The size of each frame is resized to 256*128. Random erasing for each frame and random horizontal flip for each sequence are used as data argumentation. The ResNet-50 pre-trained on ImageNet dataset is used as our backbone, and Adam is adopted with weight decay 0.0005 as the optimizer. We trained our model for 150 epochs with an initial 3.5*e*−4 learning rate and decayed by 10 at every 40 epochs.

### 4.3 Comparison with state-of-the-arts

**Figure 6:**
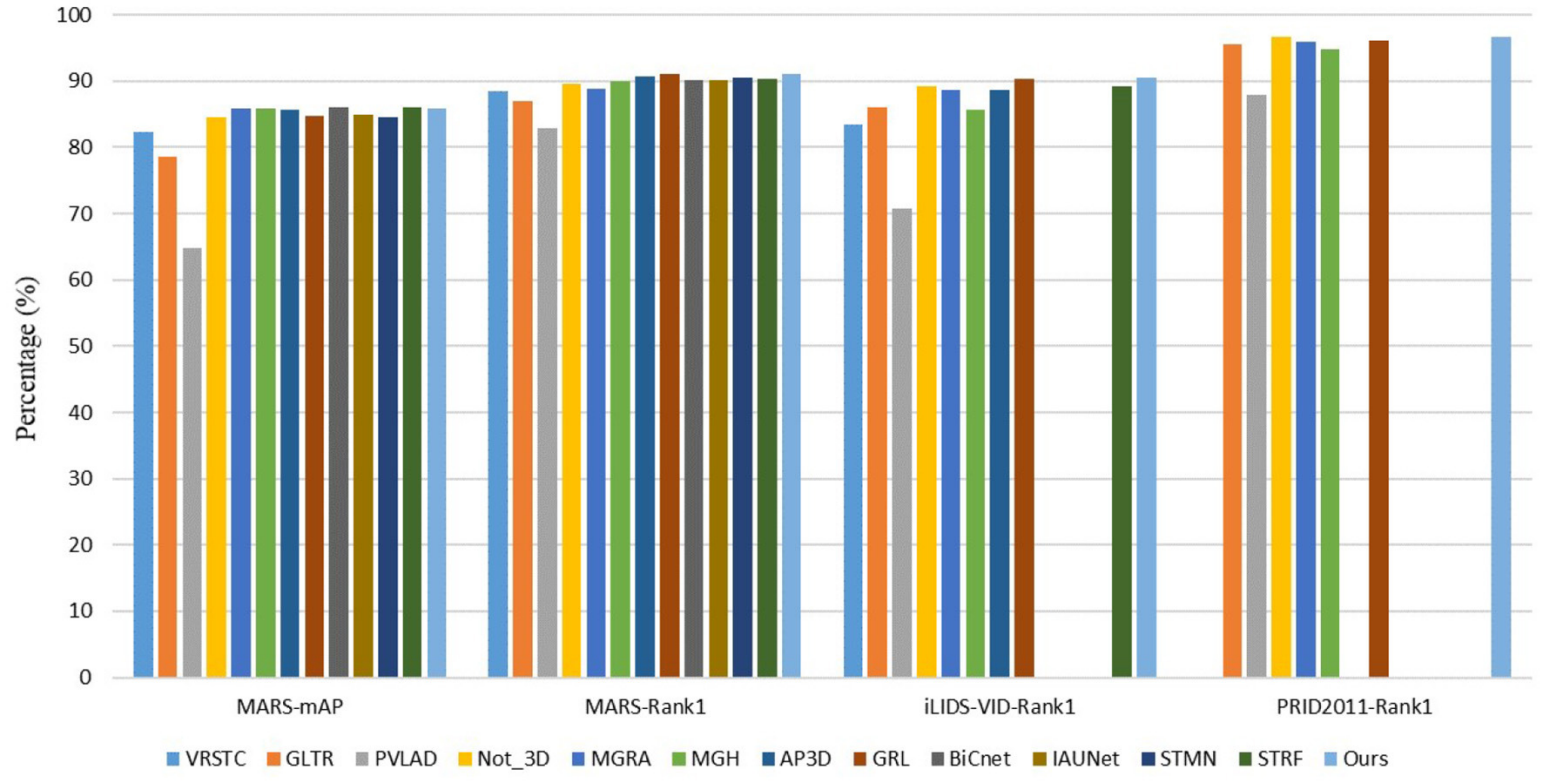
Performance (%) comparison of our method with state-of-the-art methods.

We compare our method with other state-of-the-art methods on three widely used video-based person re-identification datasets. The results are shown in Table 1 and Figure 6. It shows that our approach outperforms other existing methods.

**Table 1:** Performance (%) comparison of our method with state-of-the-art methods.

		**MARS**		**iLIDS-VID**	**PRID-2011**
**Methods**	**Source**	**mAP**	**Rank-1**	**Rank-1**	**Rank-1**
VRSTC (Hou et al., 2019)	CVPR 2019	82.3	88.5	83.4	-
GLTR (Li et al., 2019b)	ICCV 2019	78.5	87.0	86.0	95.5
PVLAD (Wu et al., 2019)	TNNLS 2019	64.7	82.8	70.7	88.0
Not_3D (Breckon and Alsehaim, 2021)	ICPR 2020	84.6	89.6	89.3	**96.6**
MGRA (Zhang et al., 2020)	CVPR 2020	85.9	88.8	88.6	95.9
MGH (Yan et al., 2020)	CVPR 2020	85.8	90.0	85.6	94.8
AP3D (Chen, 2020)	ECCV 2020	85.6	90.7	88.7	-
GRL (Liu et al., 2021)	CVPR 2021	84.8	**91.0**	90.4	96.2
BiCnet (Hou et al., 2021a)	CVPR 2021	86.0	90.2	-	-
IAUNet (Hou et al., 2021b)	TNNLS 2021	85.0	90.2	-	-
STMN (Eom et al., 2021)	ICCV 2021	84.5	90.5	-	-
STRF (Aich et al., 2021)	ICCV 2021	**86.1**	90.3	89.3	-
Ours	-	85.9	**91.0**	**90.6**	**96.6**

Bold values indicate the best result in each column.

We note that our approach has lower mAP on the MARS dataset compared with some methods such as BiCnet (Hou et al., 2021a) and STRF (Aich et al., 2021). It makes sense since BiCnet contains two branches to gather both detail clues and context clues. It also uses some attention modules and temporal convolution methods to focus on discriminative spatial clues and adaptively varies the scale of temporal modeling. For STRF, it proposed STRF module to model both spatial coarse/fine and temporal dynamic/static components for 3D CNN. Different from them, our approach only uses 2D CNN, models temporal clues from short term to long term, from low-level features to high-level features, and allows frame adjustment to reduce the influence of frame-level jitter. Thereby, our method could only use a single branch and surpass BiCnet by 0.8%, STRF by 0.7% of Rank-1 on the MARS dataset while having a similar result of mAP. Other methods mentioned in Table 1 do not either use temporal clues or use unsuitable modeling ways which cause lower results.

### 4.4 Ablation study

In this subsection, we conduct experiments to verify the effectiveness of the proposed methods, and the relevant ablation studies will be conducted on the MARS dataset. The results are shown in the following tables. All input images are resized to 256*128 and the Baseline means results conducted by backbone with cross entropy and triplet loss.

First, the ablation study of two main components of our methods is shown in Table 2. In this table, “+AA” and “+RGTCN” mean baseline only with the AA module and RGTCN module, respectively. For the AA module, since it is designed for the following modeling of temporal clues, using the AA module solely could only improve limited performance. We can see the AA module improves 0.5% mAP and 1.5% Rank-1. This result shows that the AA module could still work without dedicated temporal modeling because the final global average pooling is still a weak temporal modeling method and the AA module helps to reduce the appearance of unexpected parts in final features. For the RGTCN module, we can see a remarkable improvement in the final result. It surpasses 1.1% mAP and 1.3% Rank-1 with the baseline. This indicates that RGTCN could extract robust and discriminative features. Though some misalignment will affect the final performance, it can still achieve a good result.

**Table 2:** Ablation results of two main components on MARS.

	**MARS**	
**Models**	**mAP**	**Rank-1**
Baseline	84.5%	89.1%
+AA	85.0%	90.6%
+RGTCN	85.6%	90.4%
+AA+RGTCN	85.9%	91.0%

#### 4.4.1 Effectiveness of two different directions

We first investigate the influence of two directions for the AA module. To replace the origin module, we fuse two parts into one branch and double output channels to generate two offsets simultaneously. The results are shown in Table 3, and we can see that splitting two directions into two different branches could achieve a better result. The mAP and Rank-1 could surpass 0.2% and 0.4%, respectively, since separating directions reduce noise from another direction.

**Table 3:** Ablation results of two directions for AA Module.

	**MARS**	
**Models**	**mAP**	**Rank-1**
Baseline	84.5%	89.1%
+One Branch Direction	84.8%	90.2%
+Two Branch Direction	85.0%	90.6%

#### 4.4.2 Effectiveness of time order

We conduct some experiments to verify the effectiveness of the proposed two directions of time. As shown in Table 4, we set three directions which are only forward, only inverted, and both of them. The results show that only the forward or inverted direction can both improve the performance, but they are focusing on different parts. It seems that the forward direction focuses more on which one is the exact query identity since it achieves a better Rank-1 result, and the inverted direction focuses more on a global similarity due to its excellent mAP result. Hence, we add two directions to our module, and they perform better than only using one of them.

**Table 4:** Ablation results of two time order for RGTCN.

	**MARS**	
**Models**	**mAP**	**Rank-1**
Baseline	84.5%	89.1%
+Forward	85.3%	90.1%
+Inverted	85.6%	89.8%
+Both	85.6%	90.4%

#### 4.4.3 Effectiveness of two kinds of BN

Finally, we remove the global BN and normal BN layer separately in the GTCN module to verify whether global and local information could further improve the result, respectively. As shown in Table 5, when using global BN, it can achieve a close result compared with using both BN layers, and for only normal BN, it could also surpass the baseline but lower than global BN. This indicates that the network focuses on global clues more than specific local clues, but specific local clues could still be an auxiliary part of the final representation since the best result is achieved by using both of them.

**Table 5:** Ablation results of two different batch norm for GTCN.

	**MARS**	
**Models**	**mAP**	**Rank-1**
Baseline	84.5%	89.1%
+Normal BN	85.0%	90.0%
+Global BN	85.5%	90.3%
+Both	85.6%	90.4%

## 5 Conclusion

In this study, we propose a novel temporal modeling method named AA-RGTCN, and it has two main components. The adaptive Alignment module will address the misalignment of frames to allow a better sequence-level description. The RGTCN module is proposed to generate temporal representation which contains various features from short term to long term, from low-level features to high-level features, and two meaningful time orders. Based on these two modules, our methods could generate abundant and discriminative temporal features while conquering the misalignment problem. Extensive experiments on three public datasets show our method has state-of-the-art performance. Right now, the AA module could only shift and align frames, and it cannot restore missing parts due to frame clipping. Hence, we may still lose some discriminative features. In the future, we plan to allow the AA module to generate the missing part in the proper position based on the temporal information from other frames. We think it is an interesting method and could gain better performance.

## Data availability statement

The original contributions presented in the study are included in the article/supplementary material, further inquiries can be directed to the corresponding author.

## Author contributions

YZ: Conceptualization, Project administration, Supervision, Writing – review & editing. YL: Conceptualization, Investigation, Software, Writing – original draft, Writing – review & editing. XY: Conceptualization, Funding acquisition, Project administration, Supervision, Writing – review & editing.
